# Development and evaluation of the psychometric properties of a new measure of athlete insomnia: Insomnia in Response to Sports‐related Stress Test questionnaire

**DOI:** 10.1002/ejsc.12095

**Published:** 2024-03-18

**Authors:** Mathieu Nédélec, Maxime Chauvineau, Guillaume Martinent

**Affiliations:** ^1^ French Institute of Sport (INSEP) Laboratory Sport, Expertise and Performance (EA 7370) Paris France; ^2^ University of Claude Bernard Lyon 1 – Univ Lyon Laboratory of Vulnerabilities and Innovation in Sport (EA 7428) Faculty of Sport Sciences Lyon France

**Keywords:** arousal, competition, exercise, recovery, sleep disturbance, training

## Abstract

To develop and validate the Insomnia in Response to Sports‐related Stress Test (IRSST) questionnaire, a new specific instrument with the goal of sensitively measuring vulnerability to sport‐specific stressful situations among elite athletes. Five hundred and thirty‐one competitive elite athletes (mean age = 17.6 ± 4.4 years) completed the Ford Insomnia Response to Stress Test (FIRST) questionnaire and the IRSST, a six‐item questionnaire developed to assess the level of sleep disturbance in response to the commonly experienced sport‐specific stressful situations. A development and validation process including substantive, structural, and external stages was used in the present study. One eigenvalue of the exploratory factor analyses was greater than 1.0 (i.e., 2.91, 48.52% of explained variance) whereas the scree test provided evidence for a one‐factor solution, with all the six items achieving a loading of 0.40 or higher on the factor. Cronbach alpha was 0.77 and provided evidence for the reliability of the IRSST score. The correlation between IRSST and FIRST scores was 0.47 (*p* < 0.001, moderate effect size). These results provide strong evidence for construct validity, indicating that the IRSST is a promising scale for assessing the likelihood of sleep disruption due to sports‐related stressful situations. The results of reliability and correlational analyses provided further evidence of the promising psychometric properties of the IRSST. We believe that the IRSST could provide to the sport and sleep science communities a sleep screening tool for use in this unique population.

## Highlights

- The Insomnia in Response to Sports‐related Stress Test (IRSST) questionnaire is a promising new specific instrument for assessing the likelihood of sleep disruption due to sports‐related stressful situations
- The IRSST could provide to the sport and sleep science communities a sleep screening tool for use in the unique population of competitive elite athletes
- The IRSST can be used to identify athletes with sleep difficulties related to sports‐specific stressful situations (e.g., training, competition, injury)

## 1 INTRODUCTION

Practicing a sport at the highest level implies high training load (Dumortier et al., 2018), pressure on personal relationships, performance expectations from coaches (Botterill et al., 2002; Ommundsen et al., 2006), and constraints on personal life (Schaal et al., 2011). As a consequence, there is a high prevalence of poor sleep (49%) and poor mental health (14%–17%) in adult athletes (Drew et al., 2018). Impaired sleep, with difficulty initiating or maintaining sleep, and/or non‐restorative sleep, together with impaired daytime functioning, is particularly frequent among elite athletes (Gupta et al., 2017). Insufficient sleep has been linked to various negative consequences, including lower athletic performance (Halson, 2008) and general health (Alvarez et al., 2004). Several studies have examined potential acute and chronic factors placed on elite athletes resulting in compromised sleep, for example, training and competition (Gupta et al., 2017; Nédélec et al., 2015). Candidate risk factors for compromised sleep in elite athletes also include many factors commonly considered to influence sleep in non‐athletic individuals and particularly psychological stress and anxiety (Kalmbach et al., 2018). In a previous study, we found that susceptibility to insomnia—assessed by the Ford Insomnia Response to Stress Test (FIRST) questionnaire—may also be related to personality profile (Nédélec et al., 2021). Athletes from the maladaptive personality profile (i.e., high levels of conscientiousness and neuroticism) reported significantly higher levels of FIRST compared with those from adaptive and highly adaptive personality profiles. Higher FIRST scores were also noted for women compared with men (Aloulou et al., 2021), which suggests that women are more sensitive to sleep deterioration in response to stressful situations compared with men (Schaal et al., 2011). However, one limitation of the FIRST questionnaire is that it includes items asking about the likelihood of sleep disruption due to common stressful situations (e.g., before having to speak in public, before an important meeting the next day) but lacks information specific to athletes (Drake et al., 2004).

The aim of the present study was to develop and validate the IRSST (Insomnia in Response to Sports‐related Stress Test) questionnaire, a new specific instrument with the goal of sensitively measuring vulnerability to sport‐specific stressful situations among elite athletes. A development and validation process including substantive, structural and external stages was used in the present study based on the rationale that it provides a strong analytical framework for construct validation (Nicolas et al., 2019). The substantive stage of construct validity explores whether the content of each item captured the core of the intended construct. The structural stage provides evidence of factorial validity and reliability relative to the construct of interest. The external stage examines whether the construct under investigation is related to other variables in accordance with theoretical expectations (Messick, 1995).

## 2 METHODS

### 2.1 Participants and procedure

A total of 531 French competitive elite (Swann et al., 2015) athletes (265 men, 266 women; *M*_age_ = 17.6 years; *SD* = 4.4) participated in the study. Participants played team sports (43%: basketball, field hockey, handball, ice hockey, rugby, soccer, volleyball) or individual sports (56%: archery, artistic gymnastics, badminton, bobsleigh, boxing, sailing, cycling, diving, judo, golf, gymnastics, fencing, figure skating, horse riding, kayaking, rowing, shooting, taekwondo, track and field, synchronised swimming, swimming, table tennis, tennis, triathlon, weightlifting, wrestling; 1%: not known). They all competed at an international level. Data collection occurred once during the usual training period. Athletes completed the online questionnaire package on a computer (Tonetti et al., 2016). Instructions for completing each questionnaire were contained within the questionnaire package. Prior to participation, all athletes or a legal representative where the athlete was under 18 years old signed informed consent forms. The administration of the questionnaires met the criteria of free participation, anonymity and confidentiality of the responses. The protocol was approved by the local ethics committee (East III, France. Ref. 170605).

### 2.2 Measures

The FIRST is a standardised questionnaire that has been shown to be a sensitive measure of vulnerability to sleep disturbance and the total score has high reliability (Drake et al., 2004). The FIRST includes 9 items about the likelihood of sleep disruption due to specific stressful situations and more broadly describes periods of stress occurring during the day or evening, that is before an important meeting the next day, after a stressful experience during the day, after a stressful experience in the evening, after getting bad news during the day, after watching a frightening movie or TV show, after having a bad day at work, after an argument, before having to speak in public, before going on vacation the next day. The possible responses and corresponding scores include: *not likely* = 1, *somewhat likely* = 2, *moderately likely* = 3 and *very likely* = 4. The total score ranges from 9 to 36. High scores on the FIRST indicate greater vulnerability to sleep disruption.

Following Dunn, Bouffard and Rogers' recommendations (Dunn et al., 1999), an expert panel consisting of three researchers in the sport sciences domain was composed. Members of the panel were considered experts in questionnaire construction (i.e., already involved in and published peer‐reviewed articles related to questionnaire validation) and/or have previously conducted research on athletes' sleep disturbance. They developed a six‐item questionnaire (IRSST) to assess the level of sleep disturbance in response to the commonly experienced sport‐specific stressful situations, that is before competition, after daytime competition, after nighttime competition (20:00–21:00), after nighttime training (20:00–21:00), during training camp, after injury (Table 1). (Gupta et al., 2017; Nédélec et al., 2015; Walsh et al., 2020) The possible responses and corresponding scores include: *not altered* = 1, *somewhat altered* = 2, *moderately altered* = 3 and *very altered* = 4. The total score ranges from 6 to 24. High scores on the IRSST indicate greater vulnerability to sleep disruption.

**TABLE 1:** The Insomnia in Response to Sport‐related Stress Test questionnaire.

When you experience the following situations, how likely is it for you to have your sleep altered? Circle an answer even if you have not experienced these situations recently.			
Before competition			
1. Not altered	2. Somewhat altered	3. Moderately altered	4. Very altered
After daytime competition			
1. Not altered	2. Somewhat altered	3. Moderately altered	4. Very altered
After nighttime competition (20:00–21:00)			
1. Not altered	2. Somewhat altered	3. Moderately altered	4. Very altered
After nighttime training (20:00–21:00)			
1. Not altered	2. Somewhat altered	3. Moderately altered	4. Very altered
During training camp			
1. Not altered	2. Somewhat altered	3. Moderately altered	4. Very altered
After injury			
1. Not altered	2. Somewhat altered	3. Moderately altered	4. Very altered

### 2.3 Data analysis

The substantive stage of construct validity was explored by ensuring the initial pool of items covered the intended construct (i.e., the content of each item captured the core of the intended construct). The structural stage of the IRSST was examined through exploratory factor analysis (EFA) using FACTOR software (Lorenzo‐Seva et al., 2006). EFA was conducted on the six original items of the IRSST to discover the number of latent factors and select the items in the final version (Isoard‐Gautheur et al., 2018). The number of factors was determined through parallel analysis using exploratory robust maximum likelihood method for factor extraction with promin rotation to achieve factor simplicity (Glorfeld, 2016). Preliminary goodness of fit indexes were provided including the chi‐square (*χ* ^2^), comparative fit index (CFI), goodness‐of‐fit index (GFI), root mean square error of approximation (RMSEA), root mean square of residuals (RMSR) with their bootstrap 95% confidence interval (95%CI). For CFI and TLI, values greater than 0.90 support adequate data fit whereas 0.95 support excellent data fit (Glorfeld, 2016). For RMSEA, values smaller than 0.08 support acceptable data fit whereas 0.06 support excellent fit (Glorfeld, 2016). Then, the reliability of the IRSST score was assessed using Cronbach's alpha coefficient. A value of 0.70 or greater indicates an acceptable reliability (Glorfeld, 2016). The external stage of IRSST was examined using Pearson's correlation (*r*) between IRSST and FIRST scores. The magnitude of the correlation (*r*) was interpreted as follows: <0.10, trivial; 0.10–0.29, small; 0.30–0.49, moderate; 0.50–0.69, large; 0.70–0.89, very large; and 0.90–1.00 (Martinent et al., 2015), almost perfect. Finally, descriptive statistics were conducted to examine the effects of gender, type of sport (individual vs. team sports) and age on the IRSST score. Thus, ANOVAs with gender or type of sport as independent variable and the IRSST score as the dependent variable were conducted to examine the effects of gender or type of sport on the IRSST score. *η* ^2^ provided an index of effect size (Cohen, 1988). The correlation (*r*) between age and the IRSST score was computed to examine the relationship between these two variables.

## 3 RESULTS

One eigenvalue of the EFA was greater than 1.0 (i.e., 2.91, 48.57% of explained variance). Parallel analysis provided evidence for a one‐factor solution. Preliminary goodness‐of‐fit indices of the EFA using parallel analysis provided evidence of the structural stage of IRSST scores as goodness‐of‐fit indices reached cut‐off criterion values for an acceptable fit to the data: *χ* ^2^ = 35.14, *p* < 0.001, CFI = 0.98, 95%CI = 0.97–0.98, GFI = 0.99, 95%CI = 0.98–0.99, RMSEA = 0.076, 95%CI = 0.072–0.080, RMSR = 0.057, 95%CI = 0.040–0.071. The six items achieved a loading comprised between 0.27 and 0.81 (Table 2). Cronbach alpha was 0.77 and provided evidence for the reliability of the IRSST score. The correlation between IRSST and FIRST scores was 0.47 (*p* < 0.001, moderate effect size). The correlation between the IRSST score and age was non‐significant (*r* = 0.04, *p* = 0.39). The IRSST scores across individual and team athletes (*F* = 1.12, *p* = 0.29, *η* ^*2*^ = 0.00, *M*_*team sport*_ = 13.86 ± 3.61, *M*_*individual sport*_ = 13.50 ± 3.83) or men and women (*F* = 1.24, *p* = 0.27, *η* ^*2*^ = 0.00, *M*_*men*_ = 13.46 ± 3.99, *M*_*women*_ = 13.83 ± 3.44) were not significantly different.

**TABLE 2:** Results of exploratory factor analysis using parallel analysis.

	Exploratory factor analysis
	Factor loadings
Item 1	0.39
Item 2	0.77
Item 3	0.81
Item 4	0.78
Item 5	0.59
Item 6	0.27

## 4 DISCUSSION

The aim of the present study was to develop and validate a new specific instrument with the aim of sensitively measuring vulnerability to sport‐specific stressful situations among elite athletes, that is the Insomnia in Response to Sports‐related Stress Test (IRSST) questionnaire. The present questionnaire responds to the need to go beyond the FIRST questionnaire, which asks about the likelihood of sleep disruption due to common stressful situations but lacks information specific to athletes (Drake et al., 2004). In this respect, the IRSST was developed to assess the level of sleep disturbance in response to six commonly experienced sport‐specific stressful situations (Gupta et al., 2017; Nédélec et al., 2015; Walsh et al., 2020). The results of EFA provided strong evidence for construct validity, indicating that the IRSST is a promising scale for assessing the likelihood of sleep disruption due to sports‐related stressful situations. The results of reliability and correlational analyses provided further evidence of the promising psychometric properties of the IRSST.

Contrasting daytime physical activity and nighttime rest is important in order to adjust the body clock (Martinez‐Nicolas et al., 2014). Conversely, the practice of nighttime exercise, that is performing intense exercise with a high degree of arousal in combination with bright light exposure, has the potential to induce chrono disruptive effects on sleep (Nédélec et al., 2015). In a previous study (Aloulou et al., 2020), we found that nighttime (21:00) high‐intensity intermittent running exercise increased the polysomnographic proportion of light sleep and decreased REM sleep compared with a resting condition among well‐trained athletes, in addition to an impairment in subjective sleep quality that night. Therefore, the commonly experienced sports‐specific stressful situations of nighttime (20:00–21:00) competition and training were included in the IRSST. Future studies are required to ascertain if the psychological strain associated with nighttime competition (Juliff et al., 2018) differently disturbs sleep compared with nighttime training. Further study could investigate the effect of competition outcome (win vs. loss; success vs. failure) on the quality of subsequent night sleep. Although Roberts et al. (2022) found that women experience greater pre‐endurance‐race stress than men and their sleep duration was associated with emotional factors, results of the present study indicated that the IRSST score did not significantly differ across gender or type of sport (individual vs. team sports). It was surprising that we did not detect a gender difference in our data. Previous research has shown that rates of sleep difficulties, such as sleep dissatisfaction, are higher in women than in men (Ohayon, 2002). Overall, our findings contrast those from the insomnia literature, which have shown that the disorder is more common among women than men (Ohayon, 2002). In addition, the IRSST score was not significantly correlated to the age of the participants. This result is different from a previous study showing that poor sleep quality was related to age, with athletes over 25 years of age reporting higher Pittsburgh Sleep Quality Index (PSQI) scores than those under 20 years of age (Swinbourne et al., 2016). Discrepancies between the studies may be explained by the different ages of the participants and/or the fact that PSQI and IRSST capture different facets of sleep quality.

Other than the commonly training and competition sports‐specific stressful situations experienced by athletes in the IRSST, an item related to the experience of injury was also included as it is likely to impact sleep (Nédélec et al., 2019). To evaluate the influence of injury on quality of life and sleep in high school volleyball women, Watson et al. compared changes in quality of life and sleep duration during the season between (1) injured and non‐injured athletes and (2) injured athletes who did or did not suffer a season‐ending injury (Watson et al., 2021). Over the course of the season, no significant interactions were identified between injury and sleep duration or between season‐ending injuries and sleep duration. However, Watson et al. suggested that athletes who sustain a severe injury are at the greatest risk of significant psychological consequences and may warrant additional screening and intervention within the clinical setting to optimize their care (Watson et al., 2021). In the present study, we were not able to assess the injury history (i.e., occurrence, severity) of the participants.

Although the results of this research showed that the IRSST has acceptable psychometric properties and that it is a promising scale, this research has some limitations that are all avenues for future research. First, sleep was not objectively measured using validated actigraphy‐based methods for example, As such, future study could examine the relationship between such measures and the IRSST score. Second, the sample of athletes in the current study were representative of a particular athletic population (i.e., athletes in elite sport or training programs). To ensure the validity of the IRSST score, the present study results should be replicated among athletes of different competitive levels. Third, the present study results were achieved from correlational analyses. As a result, longitudinal designs are needed to examine in more detail the links between the IRSST score and its potential determinants and outcomes.

In conclusion, the IRSST is a promising questionnaire—evidence of validity and reliability of its score—that can be used as a first‐line tool to screen and identify athletes with sleep difficulties related to sports‐specific stressful situations (e.g., training, competition, injury) experienced by athletes. In a consensus statement (Walsh et al., 2020), researchers urged practitioners to target specific individuals in need of help and/or address specific situations for those at risk, that is situations known to compromise sleep. We believe that the IRSST could provide to the sport and sleep science communities a sleep screening tool for use in this unique population. By utilizing the proper sleep intervention recommendations, athletes can begin to reduce sleep disturbances and optimize sleep.

## CONFLICT OF INTEREST STATEMENT

The authors declare that this research was conducted in the absence of any potential conflicts of interest. The results of the present study are presented clearly, honestly and without fabrication, falsification or inappropriate data manipulation.

## Data Availability

The raw data supporting the conclusions of this article will be made available by the authors, without undue reservation.
